# Antifungal Sesquiterpenoids from *Michelia formosana* Leaf Essential Oil against Wood-Rotting Fungi

**DOI:** 10.3390/molecules27072136

**Published:** 2022-03-25

**Authors:** Chia-Che Wu, Shou-Ling Huang, Chun-Han Ko, Hui-Ting Chang

**Affiliations:** 1 School of Forestry and Resource Conservation, National Taiwan University, Taipei 106, Taiwan; r91625016@ntu.edu.tw (C.-C.W.); chunhank@ntu.edu.tw (C.-H.K.); 2 Instrumentation Center, College of Science, National Taiwan University, Taipei 106, Taiwan; shouling@g.ntu.edu.tw

**Keywords:** antifungal activity, *Michelia formosana*, sesquiterpenoids, wood-rotting fungi

## Abstract

*Michelia formosana* (Kanehira) Masamune is a broad-leaved species widespread in East Asia; the wood extract and its constituents possess antifungal activity against wood-decay fungi. Antifungal activities of leaf essential oil and its constituents from *M. formosana* were investigated in the present study. Bioassay-guided isolation was applied to isolate the phytochemicals from leaf essential oil. 1D and 2D NMR, FTIR, and MS spectroscopic analyses were applied to elucidate the chemical structures of isolated compounds. Leaf essential oil displayed antifungal activity against wood decay fungi and was further separated into 11 fractions by column chromatography. Four sesquiterpenoids were isolated and identified from the active fractions of leaf essential oil through bioassay-guided isolation. Among these sesquiterpenoids, guaiol, bulnesol, and β-elemol have higher antifungal activity against brown-rot fungus *Laetiporus sulphureus* and white-rot fungus *Lenzites betulina*. Leaf essential oil and active compounds showed better antifungal activity against *L. sulphureus* than against *L. betulina*. The molecular structure of active sesquiterpenoids all contain the hydroxyisopropyl group. Antifungal sesquiterpenoids from *M. formosana* leaf essential oil show potential as natural fungicides for decay control of lignocellulosic materials.

## 1. Introduction

Lignocellulosic materials are organic polymeric biomaterials mainly composed of cellulose, hemicellulose, and lignin. They are easily degraded by biotic factors [1,2,3,4]. Biodegradation of lignocellulosic materials is a crucial issue for its utilization and product life cycle. Among the biodegradation of lignocellulosic materials, decay fungi cause the greatest financial losses of forest products; decay fungi include brown-rot fungi, white-rot fungi, and soft-rot fungi [3,4,5,6,7]. Traditionally, wood preservatives were applied to prevent the biodegradation of lignocellulosic materials, and most commercial preservatives are inorganic metal-containing agents. However, due to a growing focus on environmental consciousness, some highly toxic preservatives have been phased out and restricted from the global market [3,8,9].

Research and development in eco-benign fungicides for lignocellulosic materials are essential to achieve the optimal utilization of the lignocellulosic resources [10,11,12]. Many plant natural products have been proven to possess effective antifungal properties, including hinokitiol, *trans*-cinnamaldehyde, liriodenine, thymol, carvacrol, etc. [13,14,15,16,17].

*Michelia formosana* (Kanehira) Masamune, Formosan Michelia, belonging to the family Magnoliaceae, is a broad-leaved tree distributed in East Asia. Ogura et al. analyzed the natural products of *M. formosana* root extract and isolated 10 sesquiterpene lactones, including michelenolide, micheliolide, compressanolide, dihydroreynosin, parthenolide, dihydroparthenolide, costunolide, lanuginolide, reynosin, and santamarine, and one alkaloid, liriodenine [18]; Wu et al. also isolated the alkaloid compound, liriodenine, from *M. formosana* wood extract [16]. *M. formosana* extracts and its constituents possess the versatile bioactivities, including antifungal, anti-inflammatory, cytotoxic activities, etc. [16,18,19]. The aims of this study were to investigate the antifungal activity of *M. formosana* leaf essential oil against wood-decay fungi and to isolate and identify the constituents which possess antifungal activity from leaf essential oil.

## 2. Results and Discussion

### 2.1. Antifungal Activities of M. formosana Leaf Essential Oil and Its Fractions

**Figure 1:**
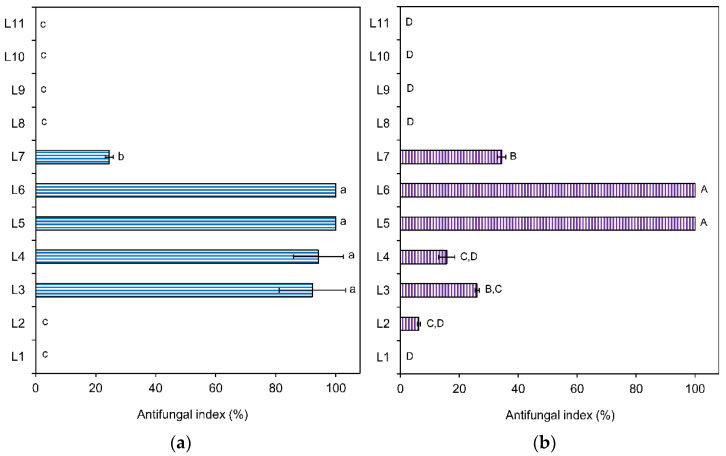
Antifungal activities of 11 fractions from leaf essential oil against wood-rotting fungi at a concentration of 200 μg/mL. (**a**) *L. sulphureus*; (**b**) *L. betulina*. Different letters (a–c; A–D) in the Figure are statistically different at *p* < 0.05 according to the Scheffe test.

Brown-rot fungi selectively degrade polysaccharides, hemicellulose and cellulose, in wood and cause the oxidation of lignin; infected wood become a brownish color due to the high residual lignin. White-rot fungi degrade both lignin and cellulosic components of wood and change the color of wood to a little whitish [3,7]. Brown-rot fungus *Laetiporus sulphureus* (*L. sulphureus*) and white-rot fungus *Lenzites betulina* (*L. betulina*; *Lenzites betulinus*; *Trametes betulina*) are common fungal strains among the wood-rotting fungi [20,21]. Antifungal indexes of *M. formosana* leaf essential oil against fungi *L. sulphureus* and *L. betulina* were 100.00% and 94.19% at a concentration of 500 μg/mL, respectively, (Table 1); 67.44% and 25.97% at a concentration of 100 μg/mL. Leaf essential oil showed a better inhibition effect against brown-rot fungus *L. sulphureus* in comparison with white-rot fungus *L. betulina*. Antifungal activity of 11 fractions of leaf essential oil against wood-rotting fungi at a concentration of 200 μg/mL are shown in Figure 1. Fractions L5 and L6 had the highest antifungal activities with an antifungal index of 100%. The other fractions showed weak/no activity against examined wood-rotting fungi.

### 2.2. Isolation and Identification of Constituents from M. formosana Leaf Essential Oil

**Figure 2:**
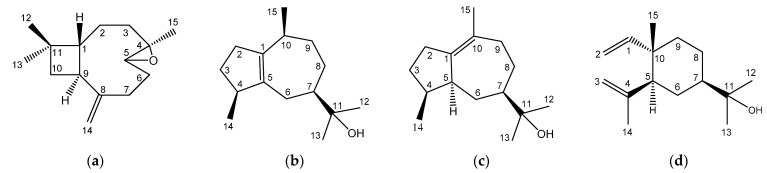
Chemical structures of sesquiterpenoids isolated from leaf essential oil. (**a**) 4,5-Epoxy-β-caryophyllene; (**b**) Guaiol; (**c**) Bulnesol; (**d**) β-Elemol.

Four sesquiterpenoids including 4,5-epoxy-β-caryophyllene, guaiol, bulnesol, and β-elemol (Figure 2) were isolated from active fractions and identified by several spectral analyses. Guaiol and bulnesol were firstly identified from woody plant *M. formosana*. Through HPLC analysis, fraction L5 contained 5.62% 4,5-epoxy-β-caryophyllene and 54.78% guaiol, and fraction L6 contained 19.54% guaiol, 54.73% bulnesol, and 13.91% β-elemol.

4,5-Epoxy-β-caryophyllene: Colorless oil, EI-MS m/z: 79, 91, 105, 121, 145, 159, 173, 187, 202. Molecular formula: C_15_H_24_O. IR ν_max_ cm^−1^: 2959 (C-H), 2920 (C-H), 1634 (C=C), 1458 (C-CH_3_) and 1383 (C-CH_3_). ^1^H NMR (CDCl_3_, 500 MHz): δ 0.94 (1H, m, H-3a), 0.98 (3H, s, H-12), 1.00 (3H, s, H-13), 1.20 (3H, s, H-15), 1.31 (1H, m, H-6a), 1.41 (1H, m, H-2a), 1.60 (1H, m, H-10a), 1.63 (1H, m, H-2b), 1.66 (1H, m, H-10b), 1.74 (1H, t, J = 10.0 Hz, H-1), 2.06 (1H, m, H-3b), 2.09 (1H, m, H-7a), 2.23 (1H, m, H-6b), 2.32 (1H, m, H-7b), 2.58 (1H, dt, J = 9.8, 9.2 Hz, H-9), 2.86 (1H, dd, J = 4.4, 10.8 Hz, H-5), 4.85 (1H, brs, H-14a), 4.97 (1H, brs, H-14b). ^13^C NMR (CDCl_3_, 125 MHz): δ_C_ 16.97 (t, C-15), 21.60 (q, C-13), 27.19 (t, C-2), 29.78 (t, C-7), 29.87 (q, C-12), 30.17 (t, C-6), 34.00 (s, C-11), 39.14 (t, C-3), 39.75 (t, C-10), 48.72 (d, C-9), 50.75 (d, C-1), 59.83 (s, C-4), 63.74 (d, C-5), 112.74 (q, C-14), 151.82 (s, C-8). 4,5-Epoxy-β-caryophyllene is a sesquiterpenoid with a structure based on the caryophyllane skeleton. NMR spectra were in agreement with the literature [22].

**Figure 3:**
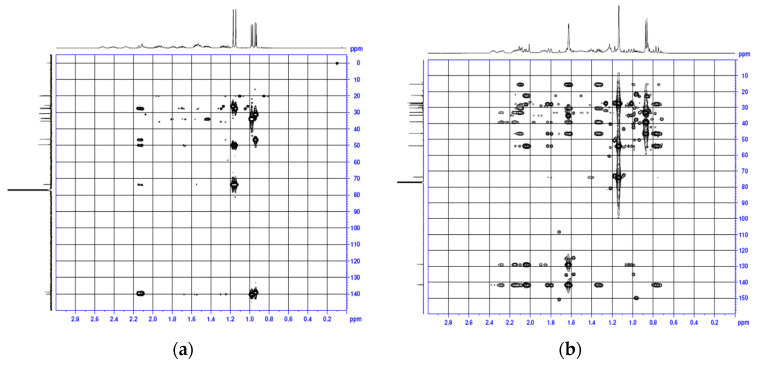
HMBC spectra of guaiol and bulnesol. (**a**) Guaiol; (**b**) Bulnesol.

Guaiol: White needles, mp: 91–93 °C. EI-MS m/z: 79, 91, 105, 119, 133, 147, 161, 189, 204, 222 [M+]. Molecular formula: C_15_H_26_O. IR ν_max_ cm^−1^: 3346 (OH), 2933 (C-H), 2856 (C-H), 1636 (C=C), 1458 (C-CH_3_), 1358 (C-CH_3_) and 918 (C-O). ^1^H NMR (CDCl_3_, 500 MHz): δ 0.91 (3H, s, H-14), 0.95 (3H, s, H-15), 1.11 (3H, s, H-12), 1.14 (3H, s, H-13), 1.25 (1H, m, H-3a), 1.42 (1H, m, H-8a), 1.51 (1H, m, H-7), 1.53 (1H, m, H-9a), 1.68 (1H, m, H-9b), 1.77 (1H, m, H-8b), 1.85 (1H, m, H-6a), 1.92 (1H, m, H-3b), 2.05 (1H, m, H-2a), 2.10 (1H, m, H-6b), 2.25 (1H, m, H-10), 2.38 (1H, m, H-2b), 2.49 (1H, m, H-4), 5.04 (1H, brs, -OH). ^13^C NMR (CDCl_3_, 125 MHz): δ_C_ 19.81 (q, C-15), 19.95 (q, C-14), 25.99 (q, C-12), 27.13 (t, C-8), 27.38 (q, C-13), 27.85 (t, C-6), 30.94 (t, C-3), 33.69 (d, C-10), 33.76 (t, C-9), 35.36 (t, C-2), 46.24 (d, C-4), 49.55 (d, C-7), 73.49 (s, C-11), 138.81 (s, C-5), 140.01 (s, C-1). Figure 3a is the HMBC spectrum of guaiol. Guaiol belongs to the guaiane skeleton which is a fused-bicyclic system with five- and seven-membered rings. NMR data of guaiol were in agreement with related literatures [23,24]. Guaiol has been reported to have antimicrobial and acaricidal activities [25].

Bulnesol (guai-1(10)-en-11-ol): Colorless oil, EI-MS m/z: 93, 105, 107, 119, 133, 161, 189, 204, 222 [M+]. Molecular formula: C_15_H_26_O. IR ν_max_ cm^−1^: 3434 (OH), 2967 (C-H), 2933 (C-H), 1632 (C=C), 1458 (C-CH_3_) and 1370 (C-CH_3_). ^1^H NMR (CDCl_3_, 500 MHz): δ 0.77 (1H, dd, J = 11.7, 24.0 Hz, H-6a), 0.87 (3H, d, J = 7.0 Hz, H-14), 1.04 (1H, t, J = 11.0 Hz, H-8a), 1.14 (6H, s, H-12,13), 1.33 (1H, m, H-3a), 1.41 (1H, m, H-7), 1.61 (1H, m, H-3b), 1.63 (3H, s, H-15), 1.81 (1H, br.d, J = 12.5 Hz, H-6b), 1.87 (1H, m, H-8b), 2.05 (1H, m, H-9a), 2.11 (1H, m, H-4), 2.13 (1H, m, H-9b), 2.15 (1H, m, H-2a), 2.29 (1H, m, H-2b), 2.37 (1H, m, H-5). ^13^C NMR (CDCl_3_, 125 MHz): δ_C_ 15.29 (q, C-14), 22.29 (q, C-15), 27.07 (q, C-13), 27.16 (q, C-12), 27.67 (t, C-8), 28.67 (t, C-6), 30.28 (t, C-2), 32.99 (t, C-3), 34.81 (t, C-9), 38.97 (d, C-4), 46.23 (d, C-5), 54.06 (d, C-7), 73.75 (s, C-11), 128.80 (s, C-10), 141.61 (s, C-1). Figure 3b is the HMBC spectrum of bulnesol. Bulnesol also belongs to the guaiane skeleton. NMR spectra were consistent with those reported in the literature [23].

β-Elemol: Light yellow oil, EI-MS m/z: 79, 93, 105, 119, 133, 147, 161, 175, 189, 204. Molecular formula: C_15_H_26_O. IR ν_max_ cm^−1^: 3424 (OH), 3083 (C=C-H), 2973 (C-H), 2936 (C-H), 2864 (C-H), 1636 (C=C), 1460 (C-CH_3_) and 1375 (C-CH_3_). ^1^H NMR (CDCl_3_, 500 MHz): δ 0.96 (3H, s, H-15), 1.18 (6H, s, H12, 13), 1.25 (1H, m, H-8a), 1.32 (1H, m, H-7), 1.40 (1H, m, H-6a), 1.42 (2H, m, H-9a, 9b), 1.56 (1H, m, H-6b), 1.63 (1H, m, H-8b), 1.69 (3H, brs, H-14), 1.94 (1H, dd, J = 12.0, 2.5 Hz, H-5), 4.56 (1H, brs, H-3a), 4.80 (1H, d, J = 1.5 Hz, H-3b), 4.86 (1H, dd, J = 11.0, 1.0 Hz, H-2 *cis*), 4.87 (1H, dd, J = 17.5, 1.0 Hz, H-2 *trans*), 5.78 (1H, dd, J = 17.5, 11.0 Hz, H-1). ^13^C NMR (CDCl_3_, 125 MHz): δ_C_ 16.57 (q, C-15), 22.53 (t, C-8), 24.77 (q, C-14), 27.13 (q, C-13), 27.15 (q, C-12), 28.47 (t, C-6), 39.69 (s, C-10), 39.85 (t, C-9), 49.32 (d, C-7), 52.68 (d, C-5), 72.75 (s, C-11), 109.88 (t, C-2), 112.03 (t, C-3), 147.89 (s, C-4), 150.22 (d, C-1). β-Elemol is an elemane-type skeleton sesquiterpenoid. NMR spectra were in agreement with those reported in the literature [23].

### 2.3. Antifungal Effect of Sesquiterpenoids from M. formosana Leaf Essential Oil

Antifungal activities of isolated sesquiterpenoids against wood-rotting fungi are presented in Table 2 below. 4,5-Epoxy-β-caryophyllene was not effective against both fungi; the other sesquiterpenoids possessed an inhibition effect with IC_50_ value less than 100 µg/mL. The compounds guaiol, bulnesol, and β-elemol showed better activities against *L. sulphureus* than against *L. betulina* comparing IC_50_ values of each specimen; the trend was similar to that of leaf essential oil, as described above. Among the three active sesquiterpenoids, bulnesol had the best inhibition effect with an IC_50_ value of 23.1 µg/mL (0.10 mM) against brown-rot fungus *L. sulphureus*. As for white-rot fungus *L. betulina*, guaiol and β-elemol were more active than bulnesol, with effective IC_50_ values of 44.1 µg/mL (0.20 mM) and 40.5 µg/mL (0.18 mM), which were lower than that of bulnesol (60.2 µg/mL; 0.27 mM). Active sesquiterpenoids belong to the guaiane and elemane-type skeletons; the molecular structure of these active compounds all contain the hydroxyisopropyl group.

Gong et al. reported that garlic essential oil and its compounds diallyl disulfide and diallyl trisulfide showed high toxicity against brown-rot fungus *L. sulphureus* with IC_50_ values of 44.6, 73.2, and 31.6 µg/mL, respectively [26]. Cinnamaldehyde is a well-known natural antifungal agent; IC_50_ values of cinnamaldehyde were 0.17 and 0.65 mM against *L. sulphureus* and *L. betulina*, respectively [11]. Wu et al. investigated antifungal activity of sesquiterpenoids from *Taiwania cryptomerioides* heartwood essential oil and derivatives against wood-rotting fungi; active antifungal compounds were α-cadinol (0.13 mM), 3β-ethoxy-T-muurolol (0.15 mM), and 15-oxo-α-cadinol (0.20 mM) against white-rot fungus *L. betulina* [27]. Present results revealed that *M. formosana* leaf essential oil and the active sesquiterpenoids, guaiol, bulnesol, and β-elemol, exhibited potent antifungal activity against wood-rotting fungi.

## 3. Materials and Methods

### 3.1. Plant Materials

Leaves of *Michelia formosana*, around 70 years old, were collected from the Experimental Forest of National Taiwan University in Nantou County, Taiwan. The voucher specimen was deposited in the Lab of Chemical Utilization of Biomaterials, School of Forestry and Resource Conservation, National Taiwan University.

### 3.2. Hydrodistillation of Leaf Essential Oil

Fresh leaves (100 g) of *M. formosana* were hydrodistilled in a Clevenger-type apparatus (1 L) with 600 mL of distilled water for 8 h to obtain essential oil [28,29,30,31]. Yield of leaf essential oil was 0.87% (*w*/*w*). The obtained leaf essential oil was stored in dark glass vials at 4 °C until used.

### 3.3. Antifungal Assay

Antifungal activity of each specimen was evaluated by using the agar plate test. The wood-rotting fungi were brown-rot fungus *Laetiporus sulphureus* Karst. (BCRC 35305, *L. sulphureus*) and white-rot fungus *Lenzites betulina* Fr. (BCRC 35296, *L. betulina*) bought from Bioresource Collection and Research Center (BCRC, Hsinchu, Taiwan). Specimens were dissolved in 90 μL (1%) of ethanol, then added into 9 mL PDA (potato dextrose agar) and mixed well in a 60 mm Petri dish. After the agar became solid, mycelial plugs (5 mm in diameter) from the edges of the blank dish were incubated in the center of each plate and cultured at 26 °C and 70% RH for 8–12 days until the fungal mycelia covered the entire control dish (1% ethanol). All experiments were repeated in triplicate. Antifungal index was calculated as the following: Antifungal index (%) = (1 − Dt/Dc) × 100, where Dt is the diameter of growth zone in the test dish and Dc is the diameter of growth zone in the control dish. IC_50_ values, half maximal inhibitory concentration, of specimens were graphically obtained from the dose response curves derived from five concentrations [16,32,33]. The positive control, didecyl dimethyl ammonium chloride (DDAC), is a commercial fungicide used in wood preservatives.

### 3.4. Bioassay-Guided Isolation by Various Chromatographies

Leaf essential oil was subjected to silica gel column chromatography (CC) with a gradient elution of *n*-hexane and ethyl acetate of increasing polarity, then separated into 11 fractions (L1-L11) by thin layer chromatography (TLC). The yields of each fraction were 35.3% (L1, elution with 100% *n*-hexane), 5.5% (L2, elution with 3% ethyl acetate/97% *n*-hexane), 11.2% (L3, elution with 5% ethyl acetate/95% *n*-hexane), 7.6% (L4, elution with 10% ethyl acetate/90% *n*-hexane), 6.9% (L5, elution with 30% ethyl acetate/70% *n*-hexane), 28.7% (L6, elution with 50% ethyl acetate/50% *n*-hexane), 2.7% (L7, elution with 50% ethyl acetate/50% *n*-hexane), 0.9% (L8, elution with 100% ethyl acetate), 0.3% (L9, elution with 100% ethyl acetate), 0.3% (L10, elution with 100% ethyl acetate), and 0.6% (L11, elution with 100% ethyl acetate). Pure compounds were obtained from active fractions by high-performance liquid chromatography (HPLC, L-2130, Hitachi, Tokyo, Japan) with a preparative 9.4 × 250 mm Zorbax Sil column (5 μm). The isocratic mobile phase consisted of *n*-hexane (90%) and ethyl acetate (10%), at a flow rate of 2 mL/min; elution peaks were detected by the refractive index (RI) detector [34,35,36].

### 3.5. Structural Elucidation

The structural determination of isolated compounds was performed by spectral analyses, including 1D NMR (Nuclear magnetic resonance spectroscopy) (^1^H-NMR, 500 MHz; ^13^C-NMR, 125 MHz) and 2D NMR (HSQC, HMBC, COSY, and NOESY) measured on a Bruker AVIII NMR spectrometer (Bruker Avance, Rheinstetten, Germany), FTIR (Fourier transform infrared spectroscopy, FTS-40, Bio-rad, Hercules, CA, USA), and MS (mass spectroscopy, MAT-958, Finnigan, MA, USA) [37,38,39,40].

### 3.6. Statistical Analysis

The Scheffe multiple comparison test of the SAS 9.3 statistical program (Cary, NC, USA) was employed to evaluate differences for the antifungal assay. The confidence interval was set at 95%.

## 4. Conclusions

Antifungal activities of *M. formosana* leaf essential oil and its constituents against wood-rotting fungi were assessed in the present study. Antifungal indexes of leaf essential oil against brown-rot fungus *L. sulphureus* and white-rot fungus *L. betulina* were 100.00% and 94.19% at a concentration of 500 μg/mL, respectively. Through the bioassay guided isolation, four sesquiterpenoids, including 4,5-epoxy-β-caryophyllene, guaiol, bulnesol, and β-elemol, were obtained from active fractions of leaf essential oil. Among the examined sesquiterpenoids, guaiol, bulnesol, and β-elemol had the best inhibition effect against wood-rotting fungi. Results indicated these sesquiterpenoids from *M. formosana* leaf essential oil have promising potential as eco-benign fungicides for decay control of lignocellulosic materials.

## Figures and Tables

**Table 1:** Antifungal index of *M. formosana* leaf essential oil against wood-rotting fungi.

Fungus	Antifungal Index (%)	
	100 μg/mL	500 μg/mL
*L. sulphureus*	67.44 ± 3.29 ^b^	100.00 ± 0.00 ^a^
*L. betulina*	25.97 ± 0.67 ^c^	94.19 ± 1.16 ^a^

Different letters (a–c) in the Table are statistically different at *p* < 0.05 according to the Scheffe test.

**Table 2:** IC_50_ values of compounds from leaf essential oil against wood-rotting fungi.

Specimen	IC_50_ (μg/mL)	
	*L. Sulphureus*	*L. Betulina*
DDAC *	0.37 ± 0.03 ^c^ ** (<0.01) ***	3.24 ± 0.11 ^C^ (0.01 ± 0.00)
4,5-Epoxy-β-caryophyllene	>100	>100
Guaiol	30.7 ± 2.8 ^a^ (0.14 ± 0.01)	44.1 ± 1.6 ^B^ (0.20 ± 0.01)
Bulnesol	23.1 ± 0.9 ^b^ (0.10 ± 0.00)	60.2 ± 2.6 ^A^ (0.27 ± 0.01)
β-Elemol	30.5 ± 2.3 ^a^ (0.14 ± 0.01)	40.5 ± 2.4 ^B^ (0.18 ± 0.01)

DDAC *: Positive control; **: Different letters (a–c; A–C) in the Table represent significantly different at the level of *p* < 0.05 according to Scheffe’s test; ***: (mM).

## Data Availability

The data are available from the corresponding author on reasonable request.

## Sample Availability

Not available.
